# Domain-Specific Acceleration of Gravity Forward Modeling via Hardware–Software Co-Design

**DOI:** 10.3390/mi16111215

**Published:** 2025-10-25

**Authors:** Yong Yang, Daying Sun, Zhiyuan Ma, Wenhua Gu

**Affiliations:** 1 School of Microelectronics, Nanjing University of Science and Technology, Nanjing 210094, China; yy@wch.cn (Y.Y.); hasdysun@njust.edu.cn (D.S.); 2 Nanjing Qinheng Microelectronics Co., Ltd., Nanjing 210012, China; 3 School of Earth Sciences and Spatial Information Engineering, Hunan University of Science and Technology, Xiangtan 411201, China; 21010103006@mail.hnust.edu.cn

**Keywords:** FPGA accelerator, gravity forward modeling, hardware–software co-design, RISC-V custom instructions

## Abstract

The gravity forward modeling algorithm is a compute-intensive method and is widely used in scientific computing, particularly in geophysics, to predict the impact of subsurface structures on surface gravity fields. Traditional implementations rely on CPUs, where performance gains are mainly achieved through algorithmic optimization. With the rise of domain-specific architectures, FPGA offers a promising platform for acceleration, but faces challenges such as limited programmability and the high cost of nonlinear function implementation. This work proposes an FPGA-based co-processor to accelerate gravity forward modeling. A RISC-V core is integrated with a custom instruction set targeting key computation steps. Tasks are dynamically scheduled and executed on eight fully pipeline processing units, achieving high parallelism while retaining programmability. To address nonlinear operations, we introduce a piecewise linear approximation method optimized via stochastic gradient descent (SGD), significantly reducing resource usage and latency. The design is implemented on the AMD UltraScale+ ZCU102 FPGA (Advanced Micro Devices, Inc. (AMD), Santa Clara, CA, USA) and evaluated across several forward modeling scenarios. At 250 MHz, the system achieves up to 179× speedup over an Intel Xeon 5218R CPU (Intel Corporation, Santa Clara, CA, USA) and improves energy efficiency by 2040×. To the best of our knowledge, this is the first FPGA-based gravity forward modeling accelerate design.

## 1. Introduction

Gravity exploration is one of the fundamental methods in geophysical prospecting. It is based on Newton’s law of universal gravitation and exploits variations in gravitational fields caused by density differences among subsurface rock and mineral bodies [1]. In geophysical exploration, gravity forward modeling refers to computing the expected geophysical response from a given geological model and its physical parameters. By applying physical equations and model attributes, forward modeling predicts the gravitational field at observation points, thereby helping to understand how different geological structures influence the measured data. This process provides a theoretical foundation for interpretation and inversion in geophysical surveys, and also offers design guidance for a range of instruments, such as MEMS-based gravimeters [2]. Most gravity forward modeling algorithms involve solving nonlinear problems [3,4]. Currently, these computations are mainly performed on CPUs [5,6]. While modern CPUs offer multi-core and multi-threading capabilities, their parallelism remains insufficient for handling large-scale, high-throughput geophysical simulations.

To enable large-scale parallel computation of gravity forward modeling, researchers have begun deploying the algorithm on GPU platforms. Compared to CPUs, GPUs can achieve over 500× speedup. However, due to the algorithm’s inherent nonlinearity and strong data dependencies, early GPU implementations primarily focused on accelerating the summation phase, which exhibits higher parallelism [7]. To further improve overall parallelism, researchers have proposed exploiting the geometric symmetry of prism integration regions to restructure the computation [8]. While this approach improves performance, it introduces accuracy loss in real-world scenarios where geological models are not perfectly symmetric. Efforts have also been made to improve portability using OpenMP  [9], enabling the algorithm to run on different platforms. However, such approaches often require algorithmic modifications to expose parallelism, limiting their applicability to specific on-site conditions. Moreover, high-performance GPUs are unsuitable for deployment in field environments due to their power consumption, size, and cooling requirements [10], making them impractical for real-time geophysical exploration and simulation tasks.

In summary, existing deployments of gravity forward modeling algorithms face two major limitations: (1) traditional CPUs struggle to meet the demands of high-performance parallel computation, and (2) the high power consumption of GPUs makes them unsuitable for on-site deployment. In remote field settings, limited access to power and communication infrastructure (e.g., no GSM signal) makes it infeasible to rely on cloud computing or large equipment [11]. However, real-time gravity modeling remains crucial for guiding exploration over complex terrain, making a lightweight, on-site solution highly desirable. Additionally, real-time local gravity modeling on remote sensing satellites [12] can greatly reduce the burden on communication bandwidth. Field Programmable Gate Arrays (FPGAs) offer a unique balance of programmability and energy efficiency. As reconfigurable hardware, FPGAs allow developers to tailor hardware circuits to specific computational tasks, making them highly adaptable. This flexibility, combined with their ability to exploit fine-grained parallelism, enables FPGAs to deliver high performance for domain-specific workloads [13], especially for scientific computing. Therefore, FPGAs have been widely adopted for specific computing architectures, including deep neural networks [14,15], quantum computing [16,17,18], sparse matrix computation [19,20], molecular docking [21,22], and so on. However, the efficient implementation of gravity forward modeling on FPGAs still faces significant challenges. In particular, the deployment of a large number of nonlinear functions on FPGA often leads to high resource utilization, increased latency, and potential numerical inaccuracies. These issues hinder the efficient deployment and practical application of gravity forward modeling on FPGA platforms.

To address these challenges, this paper proposes the first FPGA-based accelerator for gravity forward modeling. Under a hardware–software co-design framework, the accelerator begins by analyzing the parallelism of the original gravity forward modeling algorithm. A RISC-V core is integrated to retain programmability, while a set of fully pipelined processing units is designed to exploit parallel execution. Furthermore, to handle intensive nonlinear operations, we introduce a series of approximation techniques that achieve high computational efficiency with minimal precision loss. The effectiveness of the proposed design is validated across multiple datasets. The main contributions of this paper are as follows:

- We propose a custom instruction set extension for a RISC-V CPU to support an FPGA-based gravity forward modeling accelerator. To the best of our knowledge, this is the first FPGA-based accelerator design specifically targeting gravity forward modeling.
- We introduce a piecewise linear approximation method optimized using stochastic gradient descent (SGD), which significantly reduces resource utilization and computational latency. Similar approximation techniques are applied to other nonlinear operations to improve efficiency while maintaining numerical accuracy.
- We implement and evaluate our design on an AMD UltraScale+ ZCU102 FPGA.
  At a clock frequency of 250 MHz, the proposed system achieves up to 179× speedup and 2040× improvement in energy efficiency compared to an Intel Xeon 5218R processor.

The rest of this paper is structured as follows. Section 2 provides background information, focusing on the analysis of the gravity forward modeling algorithm. Section 3 presents the proposed approach, including software-level parallelism analysis, hardware-level architecture design, and corresponding optimizations. Section 4 discusses the experimental results, evaluating the accuracy and performance of the proposed accelerator. Finally, Section 6 concludes the paper and outlines directions for future work.

## 2. Background

**Figure 1:**
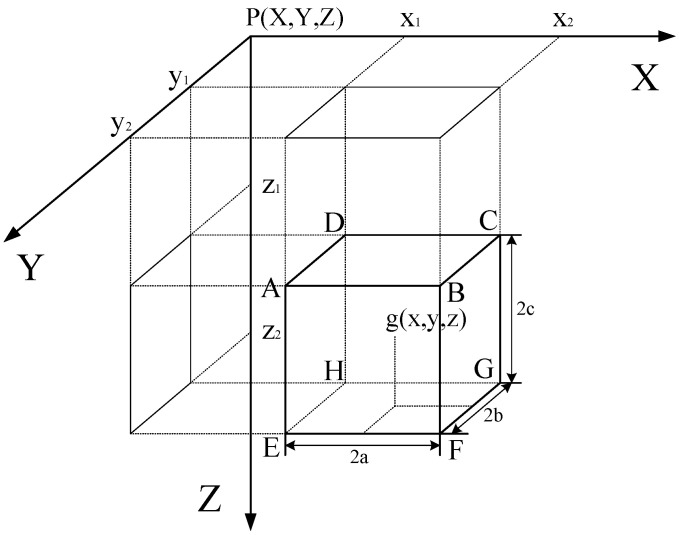
Gravity forward model.

The prism model [23] is one of the most commonly used models in gravity forward modeling due to its strong geometric adaptability, flexible parameterization, and high computational efficiency, making it well suited for 3D modeling. A typical abstraction and notation of the prism-based gravity forward model are shown in Figure 1.

In this model, the faces of the prism are aligned with the coordinate planes in a right-handed Cartesian coordinate system defined by axes X,Y, and *Z*. Let point *P* denote the observation location, and let point *g* represent the center of a geological prism ABCDEFGH, whose dimensions are 2a,2b, and 2c in the X,Y, and *Z* directions, respectively. Assuming a uniform density σ for the prism, the gravitational potential at the observation point P(X,Y,Z) can be computed according to Newton’s law of universal gravitation as follows:

(1) $$G\left(P\right)=f\sigma \underset{V}{\;\int \int \int \;}\frac{dx\;dy\;dz}{r},$$

where G(P) denotes the gravitational potential at point *P*, obtained by performing a volume (triple) integral over the region *V*, which in this case corresponds to the prism ABCDEFGH shown in the Figure 1. The $f=6.67\times 10^{-8}\;{\mathrm{cm}}^{3}/\left(g\cdot s^{2}\right)$ is the gravitational constant in CGS units, and *r* is the distance between the center of mass of the geological body and the observation point *P*, defined as:

(2) $$r=\sqrt{{\left(X-x\right)}^{2}+{\left(Y-y\right)}^{2}+{\left(Z-z\right)}^{2}}.$$

Therefore, the gravity anomaly at the observation point *P* induced by the geological body is therefore given by:

(3) $$\Delta G=f\sigma {\;\int \int \int \;}_{V}\frac{\left(z-Z\right)}{r^{3}}\;dx\;dy\;dz.$$

The earliest analytical formulation of the gravitational potential for a rectangular prism was proposed by Haaz [24], and is expressed as

(4) $$\frac{\Delta G}{f\sigma }=\left[x_{i}\ln\left(y_{j}+r_{ijk}\right)+y_{j}\ln\left(x_{i}+r_{ijk}\right)+z_{k}\arctan\left(\frac{z_{k}r_{ijk}}{x_{i}y_{j}}\right)\right]{|}_{x_{1}}^{x_{2}}{|}_{y_{1}}^{y_{2}}{|}_{z_{1}}^{z_{2}}.$$

Here, $x_{i}$, $y_{j}$, and $z_{k}$ represent the relative distances along each axis from the observation point *P* to the vertices of the prism ABCDEFGH. The distance parameters are defined as:

(5) $$\left\{\begin{array}{cc} x_{i} & ={\left(-1\right)}^{i}a+X-x,\\ y_{j} & ={\left(-1\right)}^{j}b+Y-y,\\ z_{k} & ={\left(-1\right)}^{k}c+Z-z, \end{array}\right.\;i,j,k=1,2.$$

In this expression, $r_{ijk}$ denotes the Euclidean distance from each vertex of the prism to the observation point *P*, i.e.,

(6) $$r_{ijk}=\sqrt{x_{i}^{2}+y_{j}^{2}+z_{k}^{2}}.$$

By substituting Equations (5) and (6) into Equation (4), we obtain the full expanded form of the gravitational potential equation,

(7) $$\begin{array}{cc} \frac{\Delta G}{f\sigma }= & x_{2}\ln\left(y_{2}+r_{222}\right)+y_{2}\ln\left(x_{2}+r_{222}\right)+z_{2}\arctan\left(\frac{z_{2}r_{222}}{x_{2}y_{2}}\right)\\ \; & +x_{1}\ln\left(y_{2}+r_{221}\right)+y_{2}\ln\left(x_{1}+r_{221}\right)+z_{1}\arctan\left(\frac{z_{1}r_{221}}{x_{1}y_{2}}\right)\\ \; & +x_{1}\ln\left(y_{1}+r_{112}\right)+y_{1}\ln\left(x_{1}+r_{112}\right)+z_{2}\arctan\left(\frac{z_{2}r_{112}}{x_{1}y_{1}}\right)\\ \; & +x_{2}\ln\left(y_{1}+r_{211}\right)+y_{1}\ln\left(x_{2}+r_{211}\right)+z_{1}\arctan\left(\frac{z_{1}r_{211}}{x_{2}y_{1}}\right)\\ \; & -x_{2}\ln\left(y_{2}+r_{221}\right)-y_{2}\ln\left(x_{2}+r_{221}\right)-z_{1}\arctan\left(\frac{z_{1}r_{221}}{x_{2}y_{2}}\right)\\ \; & -x_{2}\ln\left(y_{1}+r_{212}\right)-y_{1}\ln\left(x_{2}+r_{212}\right)-z_{2}\arctan\left(\frac{z_{2}r_{212}}{x_{2}y_{1}}\right)\\ \; & -x_{1}\ln\left(y_{2}+r_{122}\right)-y_{2}\ln\left(x_{1}+r_{122}\right)-z_{2}\arctan\left(\frac{z_{2}r_{122}}{x_{1}y_{2}}\right)\\ \; & -x_{1}\ln\left(y_{1}+r_{111}\right)-y_{1}\ln\left(x_{1}+r_{111}\right)-z_{1}\arctan\left(\frac{z_{1}r_{111}}{x_{1}y_{1}}\right). \end{array}$$

**Figure 2:**
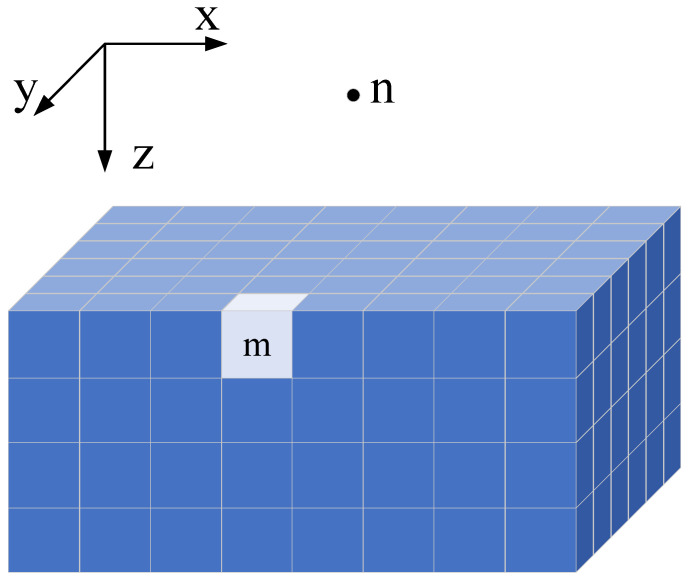
Three-Dimensional gravity forward model.

For 3D gravity forward modeling, the subsurface volume is discretized into a set of prism elements, each assumed to have a uniform density. The density values may vary across different prisms. A three-dimensional forward model can thus be constructed, as illustrated in Figure 2.

Let *M* denote the total number of prisms and *N* the number of observation points on the surface grid. According to the principle of superposition, the gravity anomaly $G_{n}$ at the nth observation point is the cumulative effect of gravitational contributions from all *M* prism elements. This can be mathematically expressed as:

(8) $$G_{n}=\sum_{m=1}^{M}\Delta G_{nm}.$$

The pseudocode for CPU-based implementation of the gravity forward modeling algorithm is shown in Algorithm 1. According to Equation (8) and Figure 2, when the surface observation grid is set to 100 × 100, the corresponding 3D subsurface model consists of 100 × 100 × 100 prism elements. This implies that on a CPU, the core computation in Equation (7) must be executed up to $10^{10}$×, highlighting the intensive computational demand of the algorithm.

For Algorithm 1, the execution begins by obtaining the model parameters defined by the user. This includes the starting coordinates of the observation plane, denoted as $P(X_{0},Y_{0},Z)$, where *Z* represents the elevation of the observation surface. The total number of observation points is given by N=L·U, where *L* and *U* denote the number of points along the horizontal and vertical directions, respectively, with a horizontal and vertical spacing of *A* and *B*. The subsurface model is discretized into a grid of rectangular prisms, starting at coordinate $m(x_{0},y_{0},z_{0})$, with a total of M=I·J·K prisms. Each prism has dimensions 2a, 2b, and 2c, and is assigned a density value $\sigma_{m}$. Next, the algorithm generates the coordinates of each observation point $\left(X_{l},Y_{u}\right)$ and each prism center $\left(x_{i},y_{j},z_{k}\right)$, and performs a five-level nested loop to compute gravitational contributions. Executing Algorithm 1 on a CPU is highly compute-intensive, due to CPUs have limited parallelism and rely on a sequential instruction-fetch-execute architecture. As a result, each gravitational calculation must be carried out in sequence, imposing a significant computational bottleneck.
**Algorithm 1:** Core Algorithm for Forward Gravity Calculation on CPU
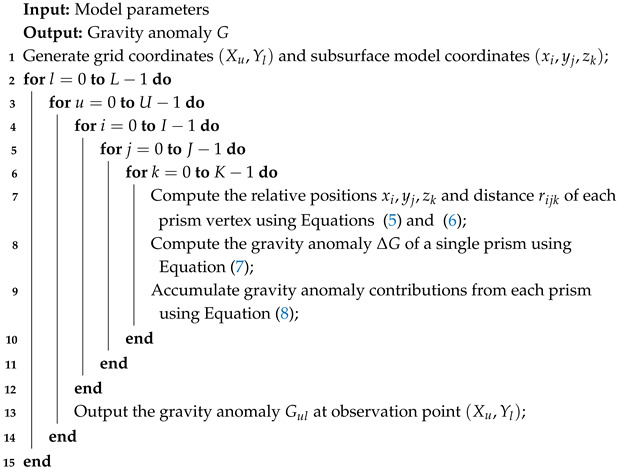



## 3. Methods

This section presents the proposed hardware–software co-design methodology for the FPGA-based gravity forward modeling accelerator. On the software side, we introduce the parallelization strategy and the design of custom instruction set extensions for the RISC-V. We then describe a piecewise linear fitting method optimized via gradient descent, as well as additional approximation algorithms for other nonlinear functions. On the hardware side, we detail the overall system architecture along with the microarchitecture design of each key module.

### 3.1. Parallelization Strategy

**Figure 3:**
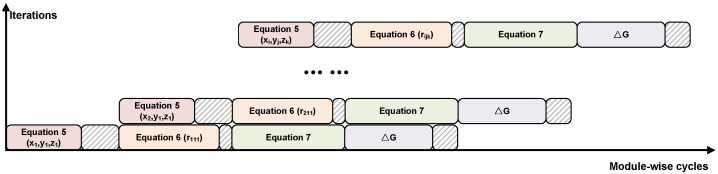
The gravity forward modeling algorithm’s computational process on FPGA.

Algorithm 1 reveals that the primary computational burden in gravity forward modeling arises from five nested loops. To map this process onto FPGA hardware, we first deconstruct the algorithm into discrete steps, analyze the data and control dependencies, and identify parallelizable operations. In our analysis, $t_{f}$ denotes the time for a single arithmetic operation on the FPGA. As illustrated in Figure 3, we provide a cycle-level view of the expected execution time, where the *y*-axis represents the total iteration count (number of executions) and the *x*-axis captures the processing timeline (execution time). Each cell reflects a unit computation cycle, which helps visualize the available parallelism.

During the first pipeline stage, the FPGA performs a parallel computation of Equation (5), completing the evaluation of the six vertex coordinates of prism ABCDEFGH in a single clock cycle. In the second cycle, the FPGA executes Equation (6) in parallel. First, it takes one unit of time to compute the squares of the six coordinate components in parallel. Then, it spends another unit of time to calculate the squared distances from the observation point to each of the eight prism vertices. Finally, five additional execution cycles are required to compute the square roots of these distances. In total, the computation of $r_{ijk}$ takes seven execution cycles. The reason the square root operation requires five execution cycles is that nonlinear functions such as square roots cannot be computed directly in hardware and must be approximated numerically. In this work, we adopt a third-order Taylor expansion to approximate the square root, as shown in follow,

(9) $$f\left(x\right)=1+\left(\frac{1}{2}\right)\left(x-1\right)-\left(\frac{1}{8}\right){\left(x-1\right)}^{2}.$$

A similar approach is used later for computing the arctangent and logarithmic functions. In the third cycle, the FPGA performs the parallel computation of all nonlinear terms in Equation (7), requiring 10 execution cycles. The method for calculating the total time follows the same principles as in the previous two cycles and is omitted here for brevity. Finally, an additional 6 execution cycles are used to sum all nonlinear terms and perform the multiplications with the density σ and gravitational constant *f*, yielding the final value of ΔG. As illustrated, the pipeline across computation modules is imbalanced. To achieve efficient pipelining both within and across modules, we adopt the longest stage as the baseline and insert BRAM-based FIFOs to buffer intermediate data accordingly. Details of this balancing approach are presented in the Gravity Anomaly Computation Module.

Unlike CPUs, FPGAs can initiate the next execution cycle without waiting for the current one to complete. This fundamental difference arises from the architectural distinctions between the two platforms. CPUs rely heavily on instruction sequences and must execute operations in a sequential manner, governed by the fetch-decode-execute cycle. In contrast, FPGAs follow a dataflow-driven architecture that is not constrained by instruction order. As a result, once a computational module completes processing, it can immediately begin processing the next data set, enabling continuous pipelined execution without waiting for prior instructions to finish.

### 3.2. Customize RISC-V Extended Instruction Design

To support coordinate-space mapping, certain preprocessing steps are required to generate the initial prism parameters based on empirical rules. Without this step, large volumes of data would need to be transferred between the CPU and FPGA. These parameters include prism density, prism indices, number and spacing of observation points, and the observation mode. By integrating a RISC-V core, users can efficiently update the initial prism configuration through C-language programming, enabling flexible and lightweight control logic on-chip. The RISC-V ISA allows for tailored instruction set extensions and accelerator design. Instruction encoding spaces and variable-length encoding make this accessible, letting developers customize processors while still using the standard ISA toolchain. Therefore, in order to efficiently control and utilize the gravity forward modeling accelerator, a dedicated set of RISC-V custom extension instructions has been designed, as show in Table 1. These instructions adopt the R-type format with the reserved custom-0 major opcode (0×0B). The funct7 field specifies the functional category of the instruction, while the operands are delivered through general-purpose registers (rs1, rs2, and rd). This mechanism enables the processor to configure all necessary geometric and physical parameters of the forward modeling process, initiate computation, and monitor execution status through a uniform instruction interface. The instruction set covers all essential modeling parameters: prism edge lengths (a,b,c), grid dimensions (I,J,K), starting coordinates (x0,y0,z0), prism identifiers and density values ($n,\sigma_{n}$), the total number of cells (*N*), initial observation points (X0,Y0,Z), grid spacing (A,B), and observation point counts (L,U). In addition, two dedicated control instructions are defined: START, which triggers the accelerator to begin computation, and POLL, which allows the processor to query the status of the accelerator in real time. With this design, the accelerator can be seamlessly integrated into a RISC-V processor pipeline. Since the hardware is fully pipelined, the CPU can continuously issue configuration and start instructions without waiting for the completion of previous tasks. This streaming mode of operation maximizes throughput and allows the accelerator to sustain near one-result-per-cycle performance once the pipeline is filled. The uniformity and simplicity of the custom instructions ensure that application developers can interact with the accelerator in the same way as with standard RISC-V instructions, providing both compatibility and ease of programming.

### 3.3. Hardware-Friendly Approximate Design

Approximate computing is a technique that introduces controlled computational inaccuracies to improve performance and reduce power consumption [25], while still meeting acceptable accuracy requirements. It has proven to be especially effective in scenarios involving large-scale data processing and computationally intensive workloads, particularly in resource-constrained environments. Piecewise approximation methods have been widely adopted in FPGA-based designs for nonlinear function evaluation [26,27,28]. However, deploying such methods on hardware often involves a trade-off between accuracy and resource utilization. Specifically, increasing the number of segments improves approximation accuracy but leads to higher FPGA resource consumption. Conversely, using fewer segments may result in significant errors when approximating certain types of nonlinear functions. To overcome the limitations of traditional linear fitting techniques for specific nonlinear functions, we leverage the stochastic gradient descent (SGD) method from machine learning to optimize the piecewise approximation of three nonlinear functions commonly used in gravity forward modeling: square root, arctangent, and logarithm, as shown in Table 2.

Note that in the derivation process, we treat *idx* as a constant and omit its corresponding gradient. For each segmentation point value $Y_{p}$, we divide the response into four parts to illustrate its stepwise behavior. When $x\in [B_{\mathrm{idx}},B_{\mathrm{idx}+1})$ and 0≤idx≤N, the output of the linear approximation unit exhibits stepwise behavior with respect to both $Y_{p}^{\mathrm{idx}}$ and $Y_{p}^{\mathrm{idx}+1}$. When $x\in (-\infty ,B_{1}]$, the output of the linear approximation unit only has stepwise behavior with respect to $Y_{p}^{0}$. When $x\in [B_{R},\infty )$, the output only shows stepwise behavior with respect to $Y_{p}^{N}$. Once all gradient information of the linear approximation unit is obtained, the parameters can be updated using SGD, as expressed by the following equation,

(10) $$\theta =\theta -\alpha \nabla f_{i}\left(\theta \right).$$

Here, θ denotes the set of parameters to be updated, such as $Y_{p}^{idx}$, $K_{1}$, $K_{2}$, and *B*, etc. The index *i* refers to the subset of parameters involved in training, i.e., $\left\{Y_{p}^{idx},K_{1},K_{2},\ldots ,B_{i}\right\}$. $\nabla f_{i}\left(\theta \right)$ denotes the gradient of the corresponding parameter θ with respect to index *i*. The scalar α is the learning rate, which controls the convergence behavior of the generated linear approximation function. A value of α that is too small results in slow convergence, while a value that is too large may cause parameter updates to be skipped or unstable, eventually preventing convergence. By using this update rule, appropriate learning steps can be selected, and the SGD process can be repeated multiple times to find an optimal piecewise linear approximation function within the target nonlinear function domain.

### 3.4. System Architecture

**Figure 4:**
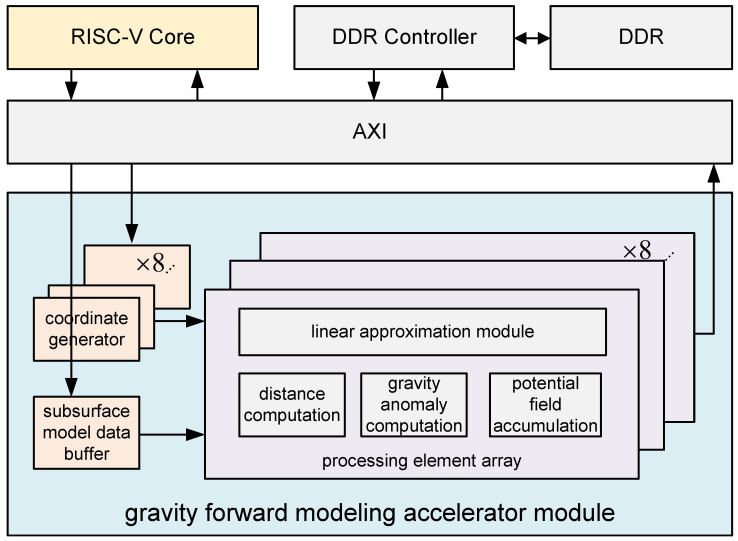
The overall hardware architecture of a gravity forward accelerator.

**Figure 5:**
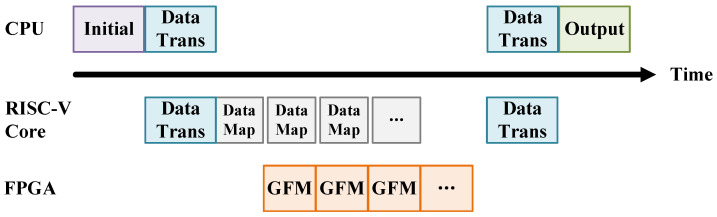
The scheduling between the CPU and accelerator system (RISC-V Core + accelerator).

The proposed gravity forward modeling hardware accelerator features an integrated architecture consisting of a RISC-V core, a DDR controller, and a dedicated computation accelerator module. All components are interconnected via the AXI bus, as shown in Figure 4. The RISC-V core is responsible for task orchestration, managing data transfers between the DDR memory and the accelerator, and handling new computation requests. It delivers the necessary input data to the accelerator, including the horizontal and vertical intervals of the observation grid, the total number of observation points along the *x*- and *y*-axes, the observation surface height *Z*, the subsurface model densities, and the prism geometry parameters. Users can control the entire accelerator system through simple C programming on a PC platform. Once programmed, the CPU (PC platform) transmits modeling-related data via UART to the RISC-V core, which decodes the data and performs mapping to generate the input format required by the accelerator, as shown in Figure 5. After the initial setup, in each subsequent computation iteration, the latency of RISC-V data mapping is fully hidden behind the accelerator’s computation, ensuring efficient pipeline utilization.

The accelerator module primarily consists of three components: subsurface model data buffers, coordinate generators, and PE (processing element) arrays. The subsurface model buffer is responsible for receiving and storing all underground prism information transferred via the system bus, including the density σ, prism dimensions *a*, *b*, and *c*, and the initial spatial coordinates $\left(x_{0},y_{0},z_{0}\right)$ of the prisms. The coordinate generator receives observation grid parameters from the decoder, along with prism dimensions and the initial coordinate $\left(x_{0},y_{0},z_{0}\right)$, and sequentially generates all required parameters for the upcoming computations—namely, the coordinates of the *j*-th observation point $\left(x_{j},y_{j},z_{j}\right)$ and the *i*-th prism center $\left(x_{i},y_{i},z_{i}\right)$. These parameters are then forwarded to the PE array, which performs gravity anomaly computations. The PE array includes a linear approximation module containing preloaded parameters for approximating the three nonlinear functions used in gravity forward modeling. It is responsible for evaluating these nonlinear functions efficiently. A distance computation module calculates the Euclidean distance *r* between each observation point and the center of each subsurface prism. The gravity anomaly computation module calculates the gravitational contribution of the *i*-th prism $\left(x_{i},y_{i},z_{i}\right)$ to the *j*-th observation point $\left(x_{j},y_{j},z_{j}\right)$. Finally, the accumulation module performs the superposition of contributions from all prisms to compute the total gravity anomaly at each observation point. The following subsections provide detailed Microarchitecture descriptions for each of these modules.

#### 3.4.1. Microarchitecture of Subsurface Model Data Buffer & Coordinate Generator

**Figure 6:**
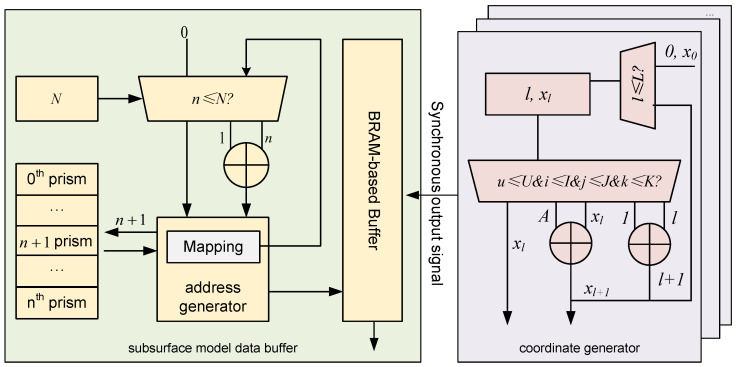
The Microarchitecture of the subsurface model buffer and the coordinate generator.

The subsurface model data buffer and coordinate generator serve as the primary data sources for the PE array, receiving data from the system bus and performing initial pre-processing. The accuracy of their computations and the synchronization of their data output directly affect the correctness of downstream calculations. The microarchitecture of the subsurface model buffer and the coordinate generator is illustrated in Figure 6.

The subsurface model databuffer is divided into three parts, which store the prism dimensions, prism density values, and the total number of prisms, respectively. The total prism count controls the address generator. When the address value reaches the total number of prisms, the address generator resets to zero, indicating that the gravity anomaly computation for the current observation point ($X_{i},Y_{i},Z$) has been completed. Otherwise, the address is incremented, and the address generator fetches the corresponding prism density from the register file. The density value is then written to a BRAM-based buffer. After the coordinate generator finishes computing the corresponding coordinates, both the spatial and physical parameters are simultaneously sent to the processing elements for gravity anomaly calculation.

The coordinate generator receives all relevant coordinate parameters and computes the spatial positions of the observation points and the subsurface prisms: specifically, $X_{l},Y_{u}$ for the observation grid, and $x_{i},y_{j},z_{k}$ for the prism centers. The update of the horizontal coordinate $X_{l}$ is controlled by the iteration counters of the four nested loops over *u*, *i*, *j*, and *k*. When all four indices reach their respective upper bounds *U*, *I*, *J*, and *K*, the index *l* is incremented by 1, and the horizontal coordinate is updated as $X_{l}=X_{l}+A$, where *A* is the spacing between observation points along the *X*-axis. If the conditions are not met, the value of $X_{l}$ remains unchanged. When l=L, it indicates that gravity anomaly computations for all observation points have been completed. At this point, the system resets l=0 and $X_{l}=X_{0}$, starting a new round of computation. Similarly, the update of the vertical coordinate $Y_{u}$ depends on the iteration status of the inner indices *i*, *j*, and *k*. When these indices reach their respective limits *I*, *J*, and *K*, the index *u* is incremented, and the vertical coordinate is updated as $Y_{u}=Y_{u}+B$, where *B* denotes the vertical spacing in the observation grid.

#### 3.4.2. Microarchitecture of PE Array

PE array is responsible for computing the gravity anomaly contribution of the *i*-th prism at a given observation point. The PE is composed of four main components: a linear approximation module, a distance computation module, a gravity anomaly computation module, and a bit-field accumulation module. The linear approximation unit stores the parameter sets for all nonlinear functions used in gravity forward modeling. These pre-loaded parameters are used by other modules to evaluate nonlinear operations efficiently. The distance computation and gravity anomaly computation modules are responsible for decomposing and computing all terms defined in Equations (5)–(7), enabling fine-grained parallelism as outlined in Section 3.1. This modular breakdown ensures each stage of the algorithm is efficiently mapped to hardware and supports pipeline execution.

**Figure 7:**
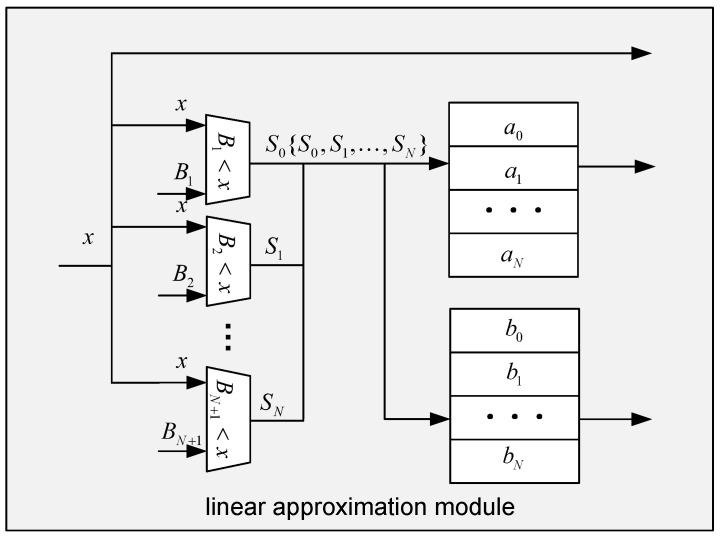
The linear approximation module’s microarchitecture.

**Linear Approximation Module:** Based on the piecewise linear approximation method proposed in Section 3.3, each of the three nonlinear functions is approximated by a set of linear segments in the form y=ax+b. To deploy these functions efficiently on FPGA, we design three customized hardware architectures, as shown in Figure 7. Each approximation unit includes multiple comparators and two lookup tables. Assuming that *N* piecewise linear segments are derived for a given function, *N* comparators are instantiated in hardware. Each comparator receives the input value *x* and compares it against the corresponding segment boundary values $B_{1}$ through $B_{N+1}$. By sequentially comparing *x* with these boundary points, the architecture determines the segment index $S_{n}$ to which the input belongs. More specifically, if $x>B_{0}$, then $S_{0}=0$; otherwise, $S_{0}=1$. This comparison process is repeated to generate the full index sequence $\left\{S_{1},S_{2},\ldots ,S_{N}\right\}$, which is then used to retrieve the corresponding segment parameters from the lookup tables. Two dedicated lookup tables are used in the linear approximation module: LUT-a stores the slopes $\left\{a_{0},a_{1},\ldots ,a_{N}\right\}$, while LUT-b stores the intercepts $\left\{b_{0},b_{1},\ldots ,b_{N}\right\}$. Once the correct index is identified, the corresponding parameters $a_{n}$ and $b_{n}$ are fetched and, together with the input value *x*, fed into a compute unit to evaluate the approximated nonlinear function value.

**Figure 8:**
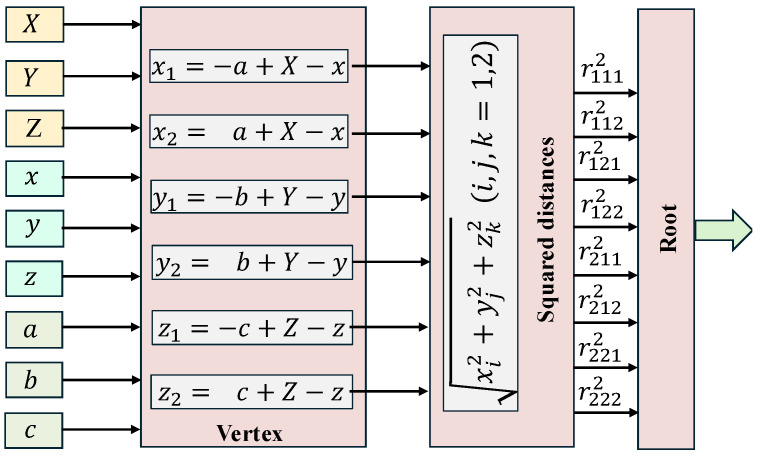
The microarchitecture of distance computation module.

**Distance Computation Module:** The distance computation module obtains the coordinates $\left(X_{n},Y_{n},Z\right)$ of the *n*-th observation point and the coordinates $\left(x_{m},y_{m},z_{m}\right)$ and dimensions *a*, *b*, and *c* of the *m*-th prism from the coordinate generator. It then enters the vertex computation unit to calculate the relative distances $x_{1},x_{2},y_{1},y_{2},z_{1},z_{2}$ between each vertex of the prism ABCDEFGH and the observation point *P* along each axis. This submodule consists of six DSP48 computing units. Next, the squared distance computation module computes the squared distances $r_{ijk}^{2}$ between each prism vertex and the observation point (i,j,k=1,2), using six multipliers. This is followed by the square root computation module, which calculates the Euclidean distance $r_{ijk}$. The square root module comprises 8 DSP48 units and approximates the square root using a piecewise linear model with slope $a_{\mathrm{sqrt}}$ and intercept $b_{\mathrm{sqrt}}$. The DSP48 is a specialized computational unit that supports fused multiply-accumulate operations among three operands. In the distance computation module, DSP48s are used in different submodules with varying configurations, as illustrated in Figure 8.

**Figure 9:**
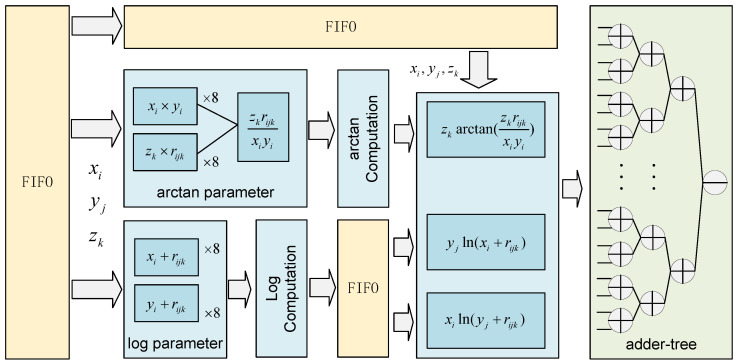
The microarchitecture of gravity anomaly computation module.

**Gravity Anomaly Computation Module:** As shown in Figure 9, the gravity anomaly computation module begins with a FIFO buffer to store the outputs $\left(x_{1},x_{2},y_{1},y_{2},z_{1},z_{2}\right)$ from previous modules. This design handles the mismatched latencies between submodules, when a computation requires results from two modules with different execution times, FIFO ensures synchronized data arrival. For example, assume the gravity anomaly module requires parameters $x_{0}$ and $r_{0}$ simultaneously. If the distance computation for $r_{0}$ takes m>n cycles while $x_{0}$ takes only *n* cycles, a mismatch occurs unless $x_{0}$ is buffered. The module would otherwise receive outdated or incorrect values such as $x_{m-n}$. Therefore, FIFO is used to preserve data order and ensure functional correctness while allowing other modules to proceed in parallel. After synchronization, data are routed to the arctangent and logarithm computation modules. The arctangent parameter computation unit includes 16 multipliers and 8 dividers, responsible for evaluating all arctangent-related terms in Equation (7). These results are then sent to the arctangent function unit, which contains 8 linear approximation engines and 8 multipliers to finalize the nonlinear function evaluations. The logarithm parameter computation module contains 16 adders, responsible for all log-related terms in Equation (7). Results are passed to the logarithm function unit, which also contains 8 linear approximation engines, 8 multipliers, and a dedicated FIFO. The FIFO aligns the output timing of the logarithmic computations with the arctangent computations, which involve fewer processing steps. Finally, the outputs from all nonlinear modules are combined to compute the gravity anomaly value. According to the sign distribution rules determined by the parity of i+j+k, each result is either added or subtracted to compute the final gravity anomaly. This design ensures the computation block can efficiently calculate the final result while preserving arithmetic correctness.

**Figure 10:**
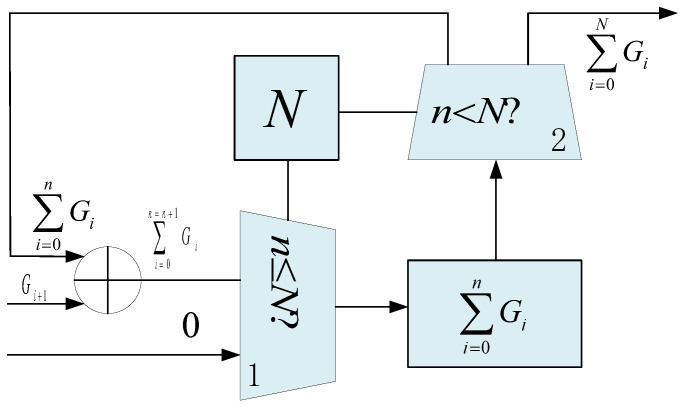
The bit-field accumulation module’s microarchitecture.

**Bit-field Accumulation Module:** The bit-field accumulation module features a relatively simple design, as shown in Figure 10. It is controlled by two selectors, governed by the total number of underground prisms *N*. The first selector determines whether the current accumulation cycle *n* is less than *N*. If n≤N, the result is valid and stored in the accumulator register. Otherwise, when n=N, the register is reset to zero. The second selector controls the timing of output. If n<N, the current accumulated result is passed to the adder for further accumulation. Otherwise, the accumulation process is considered complete and the final gravity anomaly value is output.

## 4. Results

### 4.1. Implementation Details

**Figure 11:**
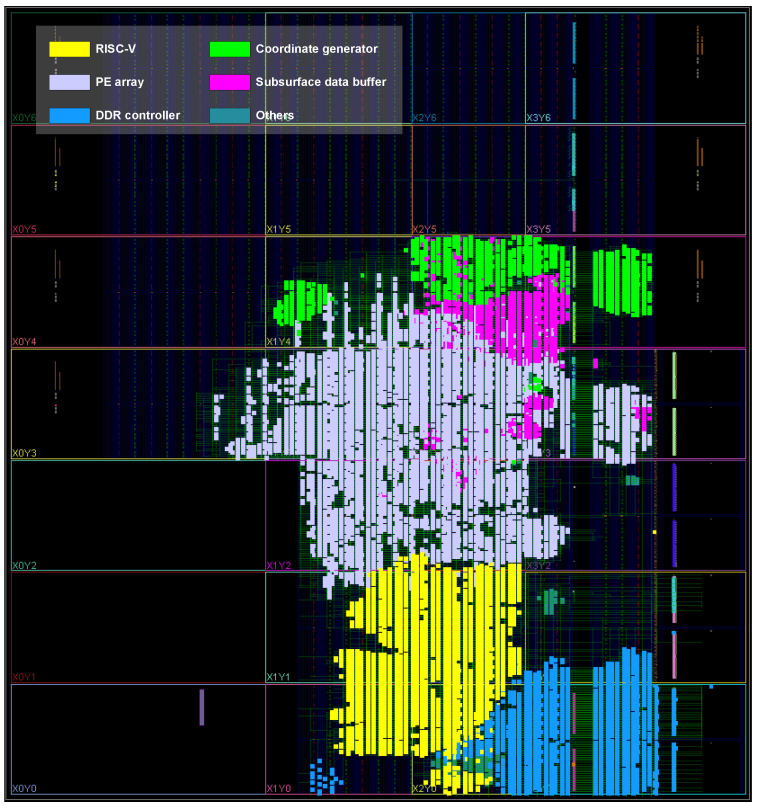
The layout of proposed accelerator design on AMD Zynq UltraScale+ ZCU102 platform.

The proposed accelerator is implemented on the AMD Zynq UltraScale+ ZCU102 platform. The entire design is developed in Verilog HDL and synthesized using AMD Vivado 2022.2. Post-implementation reports from Vivado are used to obtain resource utilization, timing, and power consumption metrics. The system operates at 250 MHz. Table 3 summarizes the resource utilization of the overall design and individual modules, and the corresponding layout is illustrated in Figure 11. The PE array is the dominant consumer of FFs (16,975) and DSPs (576), reflecting its computational intensity. The RISC-V core and coordinate generator consume notable amounts of LUTs and FFs, while the subsurface data buffer heavily utilizes BRAM (50.5 blocks). Overall, the design occupies 45,698 LUTs (16.67%), 258 LUTRAMs (0.18%), 42,310 FFs (7.72%), 170 BRAMs (18.64%), and 586 DSPs (23.25%) of the ZCU102 resources.

### 4.2. Experimental Setup

#### 4.2.1. Benchmarks

We evaluate the proposed gravity forward modeling accelerator on two benchmark models: the single-cube model [29,30], the combined -cube model [7,11], and two real-world asteroid models [31]. These experiments aim to validate both the correctness and the performance improvement of our accelerator.

For the single-cube model, we configure a 100×100 observation grid spanning a domain of X = 0∼100 km, Y = 0∼100 km, with uniform spacing of 1 km between observation points and an observation height of Z=0 km. The underground model consists of 100×100×100 prisms, covering the volume x = 0∼100 km, y = 0∼100 km, and z = 0∼−100 km, with each prism having dimensions of 1 km ×1 km ×1 km. The target anomaly is generated by a high-density cube located in the region x = 40∼60 km, y = 40∼60 km, and z = −20∼−30 km, with a density contrast of $1\;{g/\mathrm{cm}}^{3}$.

For the combined-cube model, we maintain the same 100×100 observation grid and domain settings (X = 0∼100 km, Y = 0∼100 km, Z=0 km), with 1 km point spacing. The underground model consists of 100×100×50 prisms, covering the volume x = 0∼100 km, y = 0∼100 km, z = −0.1∼−50.1 km, using the same unit prism size. The spatial distribution of the combined-cube model is summarized in Table 4.

For the asteroid models, the geometry of each asteroid is represented using a polyhedral model, which effectively captures complex surface details and supports refinement by increasing the number of vertices and faces. Polyhedral representations are also widely used in exploration geophysics to model arbitrarily complex geological targets, such as ore bodies. Therefore, the forward modeling conducted in this work is equally applicable to the gravitational field computation of complex geological structures.

#### 4.2.2. Comparison Platforms

The comparison platforms include an HPC and a HP OMEN laptop. A detailed specification comparison is provided in Table 5. The GeForce RTX4070 Laptop GPU (NVIDIA Corporation, Santa Clara, CA, USA) is chosen as a baseline to reflect realistic deployment scenarios in geological exploration, particularly in remote or mountainous regions where offline operation is required [32]. In such cases, gravity forward modeling must be performed on-site, where CPUs alone are often insufficient to meet the computational demands.

#### 4.2.3. Evaluation Index

The evaluation of the proposed accelerator focuses on two main aspects: accuracy and performance. For accuracy, gravity forward modeling results are visualized using gravity anomaly contour maps, and residual errors are computed to quantify the deviation from ground truth. For performance evaluation, we report latency, theoretical power consumption, and energy efficiency across different hardware platforms. On HPC CPU, latency is measured using system timing functions, while power consumption is monitored in real time using Intel Power Gadget. For GeForce RTX4070 Laptop GPU, we adopt reported implementations of gravity forward modeling from prior literature, and collect power consumption data using GPU-Z.

### 4.3. Accuracy Evaluation

We first evaluate the accuracy of our design by running the gravity forward modeling algorithm on both an HPC CPU (ground truth) and the proposed accelerator, using identical input parameters. The output data from both platforms are then compared for consistency. For the single-cube model, where no inter-cube interaction occurs and the computation is relatively straightforward, the results from the accelerator and the CPU are nearly identical. The maximum deviation is significantly smaller than $10^{-5}$, and therefore we omit its detailed presentation.

**Figure 12:**
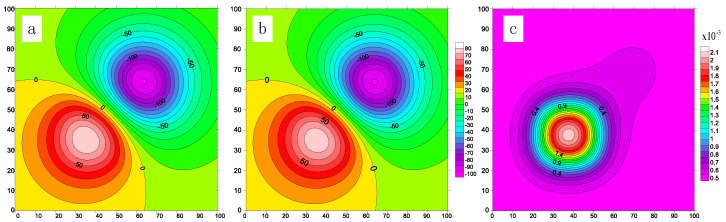
The combined-cube gravity forward modeling results: (**a**) Forward modeling result from gravity forward modeling accelerator, (**b**) forward modeling result from CPU, and (**c**) result residual.

**Figure 13:**
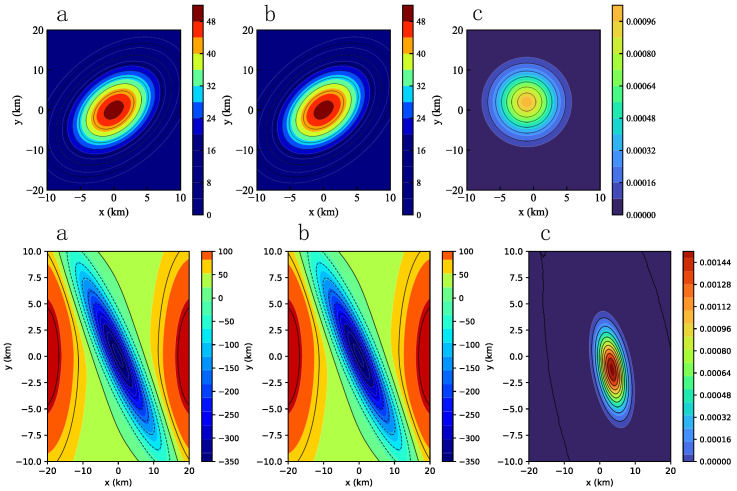
Two realworld asteroid models gravity forward modeling results: (**a**) Forward modeling result from gravity forward modeling accelerator, (**b**) forward modeling result from CPU, and (**c**) result residual.

The results for the combined-cube model are shown in Figure 12. Figure 12a presents the output of our accelerator. The gravity anomaly contours clearly align with the spatial locations of the two anomalous geological bodies, validating the spatial correlation between the anomalies and the subsurface structures. The densest contour lines correspond to the regions with the highest gravity anomalies, indicating higher densities of the subsurface bodies—consistent with physical expectations. The interaction between the two anomaly regions is also evident, demonstrating their spatial adjacency in both the X- and Y-dimensions and their overlapping gravitational influence. Figure 12b shows the results computed on the CPU. A direct visual comparison of the two plots reveals no observable differences. For completeness, Figure 12c shows the residual map, computed as the difference between the accelerator and CPU results. The maximum residual is on the order of $10^{-3}$, primarily concentrated near the boundary of the denser subsurface bodies. This small discrepancy is likely caused by non-linear approximation errors near the anomaly regions. Similarly, the gravity forward modeling results on the two real-world asteroid models are shown in Figure 13, where the residuals remain on the order of $10^{-3}$. These results also confirm that the introduced approximation techniques contribute only a negligible and acceptable level of error within the system.

### 4.4. Performance Evaluation

Table 6 presents a comparative evaluation of the proposed gravity forward modeling accelerator against two conventional platforms: an HPC (CPU) and a GPU within HP OMEN laptop. We evaluate across multiple computational loads (from $10^{5}$ to $10^{10}$ operations), power consumption, and overall energy efficiency.

Under the largest workload ($10^{10}$ operations), our accelerator achieves a latency of only 40 s, yielding 160.9× and 6.6× speedups over the CPU and GPU baselines, respectively. Even under small workloads (e.g., $10^{5}$ operations), the design maintains a significant advantage with 179.4× lower latency than the CPU.

In terms of power, the accelerator consumes only **4.36 W**, representing 12.7× and 17.7× reductions compared to the CPU and GPU. The energy efficiency benefits are even more pronounced: the accelerator delivers 57,304.60 GOPS/W, achieving a 2040× improvement over the CPU and 117× over the GPU, highlighting the suitability of the design for power-constrained geophysical modeling environments.

### 4.5. Ablation Study

**Figure 14:**
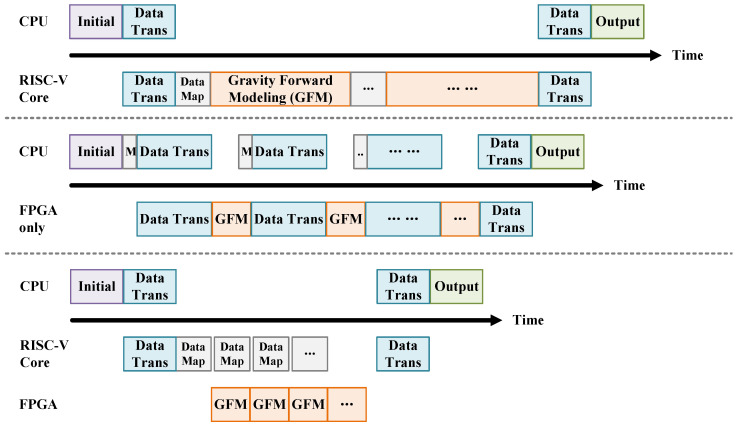
Ablation study: comparison of scheduling across different system architectures.

As described in Section 3.4, the user can control the accelerator system via a serial interface after writing a simple C program. The initial forward modeling parameters are generated directly on-chip by the RISC-V core, based on user commands. This includes horizontal and vertical intervals of the observation grid, the total number of observation points along the *x*- and *y*-axes, the observation surface height Z, the subsurface model densities, and the prism geometry parameters. If these steps were performed on a host CPU (e.g., a laptop), they would involve large volumes of data transfer to the FPGA, which would exceed the bandwidth limitations of the UART interface, as shown in Figure 14.

To clearly demonstrate the necessity of integrating RISC-V, we conduct an ablation study comparing three configurations: (1) RISC-V core only, (2) FPGA only, and (3) RISC-V core + FPGA hybrid. The soft-core serves as a programmable control plane that locally orchestrates data movement and kernel invocations, thereby mitigating host–device communication overheads. As shown in Table 7, while the standalone RISC-V core exhibits significant computational latency (56.6 s, 1.8 h, >5 days for loads of $10^{5}$–$10^{10}$), it achieves sub-millisecond communication latency due to its tight on-chip integration. In contrast, the FPGA-only design suffers from up to 5.4 min communication delay under heavy workloads, dominated by CPU–FPGA data transfers across the system bus. By co-locating the RISC-V soft-core with the accelerator logic, the hybrid system effectively eliminates most inter-device data movement. The end-to-end latency is reduced from 47.1 s to 502 ms and 6 min to 40.21 s for medium and large workloads, respectively, corresponding to ∼90× improvement in communication efficiency and ∼7× reduction in total runtime. This confirms that embedding a programmable control core near the compute fabric is a practical and efficient approach to hide control overhead and enable low-latency, self-managed FPGA acceleration.

## 5. Related Work

This work presents the first FPGA-based accelerator specifically designed for gravity forward modeling, which is a representative problem in scientific computing acceleration. In recent years, there has been a surge of research on FPGA accelerators targeting various scientific domains, with continuous architectural innovations. In the biological sciences, FPGAs have been employed for accelerating molecular dynamics simulations [22], molecular docking [34], and DNA sequence analysis and alignment [35,36]. In physical simulations, one prominent trend is the use of FPGAs to accelerate quantum computing tasks, such as quantum cryptography [18], key distillation [16], and quantum error correction [34]. In high-energy physics, FPGAs have been adopted for tasks like real-time track reconstruction [37]. More relevant to our domain, in geoscience, FPGAs have recently been applied to accelerate seismic fault interaction modeling for improved aftershock prediction [38]. As scientific computing algorithms continue to evolve and specialized hardware architectures become more accessible, FPGAs are expected to play an increasingly important role in a wide range of domain-specific scientific computing scenarios.

Approximate computing on FPGAs has gained increasing attention in recent years, with a wide range of efforts focusing on energy-efficient arithmetic designs. One of the most representative directions is the development of approximate multipliers, such as those proposed in [25,39]. For nonlinear operations, the most common approach is piecewise approximation [40,41], which requires careful trade-offs between accuracy and hardware cost. To address this, DIF-LUT [28] combines piecewise linear interpolation and table lookup to balance precision and resource usage. Similarly, Yang et al. [42] propose an enhanced Piecewise Linear (PWL) fitting algorithm for efficient approximation of nonlinear functions on FPGAs. Their method divides the domain into multiple intervals, each approximated by a linear function $f_{i}\left(x\right)=k_{i}x+b_{i}$, with parameters $k_{i}$ and $b_{i}$ derived using Cramer’s rule. Other studies focus on specific nonlinear functions, for example, Xu et al. [43] approximate the exponential function using a simplified linear transformation, preserving acceptable accuracy while reducing hardware cost. However, these approaches typically require dedicated approximation modules or custom LUT structures for each target function. In contrast, an SGD-based optimization framework can be used to unify the generation of piecewise linear parameters across different functions. This enables reuse of hardware structures, where only the function-specific parameters need to be reloaded, and support for online updates or reconfiguration, enhancing scalability and flexibility.

## 6. Conclusions

In this work, we present a domain-specific FPGA-based accelerator for gravity forward modeling, targeting the computational bottlenecks inherent in traditional CPU- and GPU-based implementations. Through a hardware–software co-design approach, we integrate a custom RISC-V processor with specialized pipelined processing elements, optimized for the high arithmetic intensity and nonlinear nature of gravitational field simulations.

To address the high resource cost of nonlinear operations such as square roots, arctangent, and logarithms, we propose a stochastic gradient descent-based piecewise linear approximation strategy, which reduces hardware complexity while maintaining acceptable accuracy. Experimental results demonstrate that our accelerator achieves up to 179× speedup and 2040× improvement in energy efficiency over a high-performance Xeon CPU, while outperforming a GPU (GeForce RTX4070 Laptop GPU) by 117× in energy efficiency. These gains make our design highly suitable for real-time, in-situ geophysical applications where power and latency are critical constraints.

Future work will explore adaptive precision strategies and partial reconfiguration techniques to further improve scalability across different gravity modeling resolutions and geological scenarios.

## Figures and Tables

**Table 1:** RISC-V Instruction for Geophysical Computation Parameters.

funct7 (7 Bits)	RISC-V Register Mapping	Semantic Meaning (Original 4-Bit Opcode)
0 × 0F (1111)	rs1 = a, rs2 = b, rd = c	Prism edge lengths (*a*, *b*, *c*)
0 × 0E (1110)	rs1 = I, rs2 = J, rd = K	Grid dimensions along x, y, z (*I*, *J*, *K*)
0 × 0C (1100)	rs1 = x0, rs2 = y0, rd = z0	Grid starting coordinates ($x_{0}$, $y_{0}$, $z_{0}$)
0 × 08 (1000)	rs1 = n, rd = $\sigma_{n}$	Prism ID (*n*) and density ($\sigma_{n}$)
0 × 00 (0000)	rs1 = N	Total number of model cells (*N*)
0 × 01 (0001)	rs1 = X0, rs2 = Y0, rd = Z	Initial observation point ($X_{0}$, $Y_{0}$, *Z*)
0 × 02 (0010)	rs1 = A, rd = B	Grid spacing along x and y (*A*, *B*)
0 × 04 (0100)	rs1 = L, rd = U	Number of observation points (*L*, *U*)
0 × 7F (extended)	no operand	Start computation (START)
0 × 7E (extended)	rd = status	Poll accelerator status (POLL)

**Table 2:** Gradients of piecewise linear unit.

∇f(θ)	$x<B_{L}$	$B_{L}\leq x<B_{R}$	$x\geq B_{R}$
$\frac{\partial U_{N}}{\partial x}$	$K_{L}$	$K_{\mathrm{idx}}$	$K_{R}$
$\frac{\partial U_{N}}{\partial B_{L}}$	$-K_{L}$	$K_{\mathrm{idx}}\cdot \frac{x-B_{R}}{B_{R}-B_{L}}$	0
$\frac{\partial U_{N}}{\partial B_{R}}$	0	$K_{\mathrm{idx}}\cdot \frac{B_{L}-x}{B_{R}-B_{L}}$	$-K_{R}$
$\frac{\partial U_{N}}{\partial K_{L}}$	$x-B_{L}$	0	0
$\frac{\partial U_{N}}{\partial K_{R}}$	0	0	$x-B_{R}$
$\frac{\partial U_{N}}{\partial Y_{p}^{\mathrm{idx}}}$	0	$\frac{B_{\mathrm{idx}+1}-x}{d}$	0
$\frac{\partial U_{N}}{\partial Y_{p}^{\mathrm{idx}+1}}$	0	$\frac{x-B_{\mathrm{idx}+1}}{d}$	0
$\frac{\partial U_{N}}{\partial Y_{p}^{0}}$	1	0	0
$\frac{\partial U_{N}}{\partial Y_{p}^{N}}$	0	0	1

**Table 3:** FPGA resource utilization of proposed accelerator.

	RISC-V	Coordinate Generator	Subsurface Data Buffer	PE Array	Others	Total
LUT	15,010	5126	2813	14,417	8332	45,698 (16.67%)
LUTRAM	34	15	20	0	189	258 (0.18%)
FF	5763	4842	4817	16,975	9913	42,310 (7.72%)
BRAM	14.5	13.5	50.5	66	25.5	170 (18.64%)
DSP	3	4	0	576	3	586 (23.25%)

**Table 4:** Geometric parameters of two cubes.

Model	*x* Range	*y* Range	*z* Range	Density Contrast
Cube 1	25∼50 km	25∼50 km	12.6∼25.1 km	$1\;g/{\mathrm{cm}}^{3}$
Cube 2	50∼75 km	5∼75 km	12.6∼37.6 km	$1\;g/{\mathrm{cm}}^{3}$

**Table 5:** Specifications of platforms.

Platforms	Processor	Frequency	On-Chip Memory
HPC (CPU)	Xeon Gold 5218R	4.0 GHz	27.5 MB L3 cache
HP OMEN laptop (GPU)	GeForce RTX4070 Laptop GPU [33]	1.39 GHz	32 MB L2 cache
Zynq UltraScale+ ZCU102	XCZU9EG	250 MHz	4.75 MB (BRAM + URAM)

**Table 6:** Performance and energy comparison of CPU and GPU.

Evaluation Index		HPC CPU (Baseline)	HP OMEN Laptop (GPU)	Ours	Improvement Over CPU	Improvement Over GPU
Latency under different computational loads	$10^{5}$	732 ms	88.7 ms	4.08 ms	179.41×	21.74×
	$10^{8}$	63.96 s	2.56 s	0.42 s	152.29×	6.10×
	$10^{10}$	6436 s	264.6 s	40 s	160.90×	6.62×
Power (W)		55.34	77.4	4.36	12.69×	17.75×
Energy efficiency (GOPS/W)		28.09	488.56	57,304.60	2040.22×	117.30×

**Table 7:** Ablation on latency comparison of system architecture variants.

	RISC-V Core Only	FPGA Only	RISC-V Core + FPGA
**Latency of communication** (computational loads $10^{5}$, $10^{8}$, and $10^{10}$)	16 ms, 82 ms, 205 ms	1.67 s, 46.7 s, 5.4 min	16 ms, 82 ms, 205 ms
**Latency of computation** (computational loads $10^{5}$, $10^{8}$, and $10^{10}$)	56.6 s, 1.8 h, >5 days	3.59 ms, 407.7 ms, 38.8 s	4.08 ms, 420 ms, 40 s
**End-to-end latency** (computational loads $10^{5}$, $10^{8}$, and $10^{10}$)	56.6 s, 1.8 h, >5 days	1.67 s, 47.1 s, 6 min	20.08 ms, 502 ms, 40.21 s

## Data Availability

The data presented in this article are available within the text. Additional data can be requested from the corresponding author.

## Abbreviations

The following abbreviations are used in this manuscript:

AXI	Advanced eXtensible Interface
BRAM	Block RAM
CGS	Centimeter–Gram–Second (unit system)
CPU	Central Processing Unit
DDR	Double Data Rate (memory)
DSP	Digital Signal Processing slice
DSP48	Xilinx DSP48 slice
FF	Flip-Flop
FIFO	First-In, First-Out (buffer)
FPGA	Field-Programmable Gate Array
GHz	Gigahertz
GOPS/W	Giga Operations Per Second per Watt
GPU	Graphics Processing Unit
HDL	Hardware Description Language
HPC	High-Performance Computing
ISA	Instruction Set Architecture
LUT	Look-Up Table
LUTRAM	Look-Up Table RAM
MHz	Megahertz
OpenMP	Open Multi-Processing
PE	Processing Element
RISC-V	Reduced Instruction Set Computer–Five
URAM	UltraRAM
